# Strategies to Improve Sensitivity to Radiotherapy in Pediatric Solid Tumors

**DOI:** 10.3390/cancers18132103

**Published:** 2026-06-29

**Authors:** Maryam G. Shaikh, Morgan L. Brown, Jamie M. Aye, Elizabeth A. Beierle

**Affiliations:** 1 Division of Pediatric Surgery, Department of Surgery, University of Alabama at Birmingham, Birmingham, AL 35233, USA; mgshaikh@uabmc.edu (M.G.S.); mlbrown6@uab.edu (M.L.B.); 2 Division of Hematology Oncology, Department of Pediatrics, University of Alabama at Birmingham, Birmingham, AL 35233, USA; jamieaye@uabmc.edu

**Keywords:** radiotherapy, radioresistance, radiosensitizers, DNA damage, pediatric cancer

## Abstract

The primary goal in the treatment of pediatric malignancies is to maintain effective cancer control while minimizing toxicities and late effects. Radiotherapy is a component of standard therapy against many pediatric cancers, but tumor cell DNA damage repair mechanisms and the hypoxic tumor microenvironment may limit the effectiveness of this modality. Additionally, irradiated cancer cells often acquire resistance to radiotherapy that permits cancer cell survival or stimulates tumor regrowth after treatment, further limiting the efficacy of radiotherapy. Various preclinical studies have investigated the potential of combining chemotherapy or immunotherapy with radiotherapy, with the goal of inducing a more lethal amount of DNA damage and prohibiting cells from acquiring radiotherapy resistance. This review provides an overview of the current strategies to enhance cancer cell response to radiotherapy, providing a framework to improve outcomes for children with solid tumors.

## Simple Summary

Radiotherapy is an important treatment for many childhood solid tumors, but it may result in serious long-term effects in children, including issues with learning, growth, hormone function, and the development of secondary cancers. Some tumors become resistant to radiation, reducing treatment efficacy and increasing the risk of tumor recurrence. This review discusses current research focused on improving tumor sensitivity to radiotherapy while limiting harmful side effects. Promising strategies include targeted drugs, immune-based therapies, DNA repair inhibitors, nanoparticle approaches, and novel radiation techniques such as FLASH radiotherapy. These emerging combination therapies have the potential to improve tumor control, reduce radiation-related toxicities, and support safer, more effective treatments for children with solid tumors.

## 1. Introduction

Pediatric solid tumors represent a diverse group of malignancies that account for a significant proportion of childhood cancers. Despite advances in multimodal treatment strategies including surgery, chemotherapy, and radiotherapy, solid tumors remain a leading cause of cancer-related morbidity and mortality in children. Radiotherapy has long been a cornerstone of treatment for many pediatric solid tumors such as central nervous system (CNS) tumors, neuroblastoma, rhabdomyosarcoma, Ewing sarcoma, and Wilms tumor, offering local control and improving survival [1,2].

Radiotherapy is a unique modality in that the dose delivered to the tumor is relatively uniform, typically within a few percent of the prescribed dose, which contrasts with chemotherapies, for which the dose delivered is heterogeneous [3]. Moreover, modern radiotherapy delivery techniques, such as stereotactic body radiotherapy (SBRT) [4], intensity-modulated radiotherapy (IMRT) [5], proton beam therapy (PBT) [6,7], and FLASH radiotherapy (FLASH-RT) [8], allow for the precise delivery of high doses of radiation directly to the tumor, especially those located near vital organs or structures. This precision is particularly advantageous in pediatric patients, whose normal tissues are developing and who are more susceptible to radiotherapy-induced damage. In addition, the systemic impact of radiotherapy is generally much lower than that of chemotherapy, making radiotherapy a suitable option for children who may require multiple rounds of treatment.

In spite of these benefits, radiotherapy in young children remains challenging due to the risk of long-term toxicities, such as growth impairment, neuroendocrine and neurocognitive deficits, and secondary malignancies [9,10,11]. The development of late effects is directly correlated with the dose delivered and the volume of tissue irradiated, and inversely correlated with patient age [12]. These limitations oblige researchers to explore options such as combination therapies that enhance the efficacy of radiotherapy while minimizing its adverse and long-term effects. These optimization strategies may include identifying predictive biomarkers to select patients who are most likely to benefit from specific combinations, understanding the mechanisms of resistance to select logical combinatorial therapies, and managing the unique toxicities associated with combined modalities. As advanced radiation delivery techniques become increasingly utilized in pediatric oncology, additional investigations will be necessary to determine whether currently identified radiosensitization strategies demonstrate comparable efficacy and toxicity profiles across different radiation modalities. Many radiosensitizers target the fundamental cellular responses to radiation-induced DNA damage and may therefore be applicable to photon or proton-based therapies; however, additional studies will be needed to define these interactions.

Newer combinations being explored to enhance therapeutic efficacy of radiotherapy include molecularly targeted agents, immune checkpoint inhibitors, and epigenetic modulators [13]. Immunotherapy may be one of the most exciting developments in combination radiotherapy [14]. The ability of ionizing radiation to induce a systemic immune response, abscopal effect, and to reprogram the tumor microenvironment, radscopal effect [15], has opened new avenues for combining radiotherapy with immunomodulators, such as anti-PD-L1 [16] and anti-CTLA-4 antibodies [17]. Preclinical [18] and early clinical studies [19] have shown promising results with these combinations in pediatric solid tumors, particularly those tumors with a high mutational burden or that are traditionally resistant to conventional therapies [20].

This review aims to provide an update on the current landscape of combinations designed to improve radiotherapy responses in pediatric solid tumors. We will discuss the rationale behind combining radiotherapy with various therapeutic modalities, highlight recent preclinical and clinical advancements, and explore emerging strategies.

## 2. Radiotherapy in Pediatric Solid Tumors

### 2.1. Radiotherapy-Induced DNA Damage

Radiotherapy uses high-energy ionizing radiation, such as X-rays, gamma rays, or charged particles, and induces DNA damage either directly or indirectly, through multiple mechanisms. The first mechanism is by direct assault. Ionizing radiation induces single-strand breaks (SSBs) or double-strand breaks (DSBs) in the DNA molecule [21]. SSBs are easier for the cell to repair but DSBs are far more lethal, as they result in chromosomal aberrations if unrepaired or repaired incorrectly. Indirectly, ionizing radiation interacts with water molecules in the cell, causing radiolysis of water generating reactive oxygen species (ROS). ROS are highly reactive and damage DNA through multiple mechanisms, including the formation of adducts that impair base pairing, blocking DNA replication and transcription, base loss, or induction of DNA SSBs. The process for repairing DNA damage is complex. Once the cell detects DNA damage through ataxia-telangiectasia mutated (ATM) and Rad3-related (ATR) protein kinases, a phosphorylation cascade is triggered, which activates the DNA damage response (DDR) pathway [22]. DDR activates checkpoint kinases (CHK1 and CHK2), which stall cell cycle progression to allow time for DNA to repair. The cell is paused in the G1, S, or G2 phase, depending on the extent of damage [23]. The DNA damage is repaired by base excision repair (BER), nucleotide excision repair (NER), non-homologous end joining (NHEJ), or homologous recombination (HR) [24]. If the damage is irreparable, the cell activates apoptosis to prevent propagation of damaged DNA. This process is controlled by tumor suppressor proteins such as p53, which sense excessive DNA damage and trigger cell death pathways. Radiotherapy exploits the cancer cell’s ability to repair DNA damage. Most cancer cells have impaired DNA repair mechanisms and high proliferation, making them susceptible to damage during DNA replication. If the DNA damage is too extensive or repair mechanisms fail, cells will not replicate properly, leading to mitotic catastrophe, senescence, or apoptosis [25]. Some tumors develop resistance to radiation by upregulating DNA repair mechanisms or activating these survival pathways [26].

Tumors with mutations in DNA damage repair genes or high levels of replication stress have an increased reliance on certain DNA damage repair pathways for maintaining cell viability [26]. Inhibition of the DDR provides a therapeutic vulnerability in tumors harboring these mutations. DDR inhibitors have shown clinical promise in adult malignancies [27]. Pediatric cancers, including neuroblastoma [28], rhabdomyosarcoma [29], and Ewing sarcoma [30], have been studied to exhibit mutations or dysregulated expression in the DDR pathway genes, which confer a proliferative advantage to these tumors. A better understanding of DDR pathway genes as potential therapeutics may pave the way for improving treatment in pediatric cancers that are resistant to conventional treatment [31].

### 2.2. Radiotherapy and Immune Response

There is ongoing research investigating the relation between radiotherapy, the DDR, and the immune response in cancer [32]. Studies have demonstrated that radiotherapy enhances MHC class I expression in a dose-dependent manner across many tumor types [33,34]. Tumors such as neuroblastoma [35] and medulloblastoma [36] exhibit reduced MHC expression to evade CD8 T-cell mediated cytotoxicity. By increasing MHC expression, radiotherapy may sensitize tumor cells to immune checkpoint inhibitors. Therefore, radiotherapy may have an immunogenic effect due to the direct presentation of tumor antigens and interferon-dependent T-cell activation [37,38]. Evidence indicates that radiotherapy may utilize the host immune system in this manner for distant tumor control via the abscopal effect [39].

Radiotherapy has also been shown to activate immunogenic cell death (ICD) [39] characterized by cell surface translocation of calreticulin, high-mobility group box 1 (HMGB1) release, and adenosine triphosphate (ATP) release. ICD secondary to radiotherapy was found to increase dendritic cell phagocytosis of tumor cells and increase the secretion of pro-inflammatory cytokines, promoting the cross-priming of CD8 T-cells [37]. Additionally, previous work suggests that radiotherapy plays a role in driving an antitumor immune response, and, in combination with immune checkpoint inhibitors, may enhance antitumor immunity [39]. Immunotherapy is a successful component of a multimodal approach to cancer and encompasses a variety of treatments, including the use of cytokines, targeted monoclonal antibodies, or molecules that inhibit immune checkpoints [40]. Currently, there are combination therapies utilizing immunotherapy and radiotherapy in many adult cancers, but this combination is less common in pediatric cancers.

### 2.3. Mechanisms of Radiotherapy Resistance in Pediatric Solid Tumors

Approximately 30% of pediatric cancer patients will receive radiotherapy as a component of their treatment [41] but radiation resistance provides a considerable challenge and determining factors that contribute to this resistance are critical to overcoming this treatment barrier [42]. The primary mechanisms by which radiation resistance occur are through pro-survival pathways including suppression of apoptosis, induction of cell cycle arrest, and promotion of DNA damage repair. These mechanisms act in tandem to promote the survival of cancer cells following radiotherapy and subsequent development of radioresistance [43].

### 2.4. Radiosensitizing Strategies in Pediatric Solid Tumors

Cancer cells often develop resistance to radiotherapy by increasing their DNA repair efficiency, typically through upregulation of DDR components. Additionally, radiotherapy-induced tissue damage may activate mitogenic signaling pathways, promoting tumor cell proliferation and repopulation. The extent of DNA damage is key to the therapeutic effectiveness of radiotherapy, and compounds that amplify the effect of radiotherapy are called radiosensitizers. Radiosensitizers may enable the use of lower radiation doses to achieve comparable antitumor effects while minimizing damage to healthy tissues and decreasing the risk of long-term effects. Additionally, the risk of added toxicity of such combination therapies is lower, as the cytotoxic effect of a successful radiosensitizer is largely confined to the irradiated tumor avoiding normal tissues. Finally, radiosensitizers may be easily integrated into standard clinical protocols, complementing the existing radiotherapy regimens. However, radiosensitization may be a complicated process. Sufficient levels of oxidative stress or DNA damage are still required to achieve the desired antitumor effects. Therefore, it is important to highlight personalized and stratified approaches instead of using a single radiosensitizer across an unstratified patient population, which has been the predominant strategy in most clinical trials to date.

**Figure 1:**
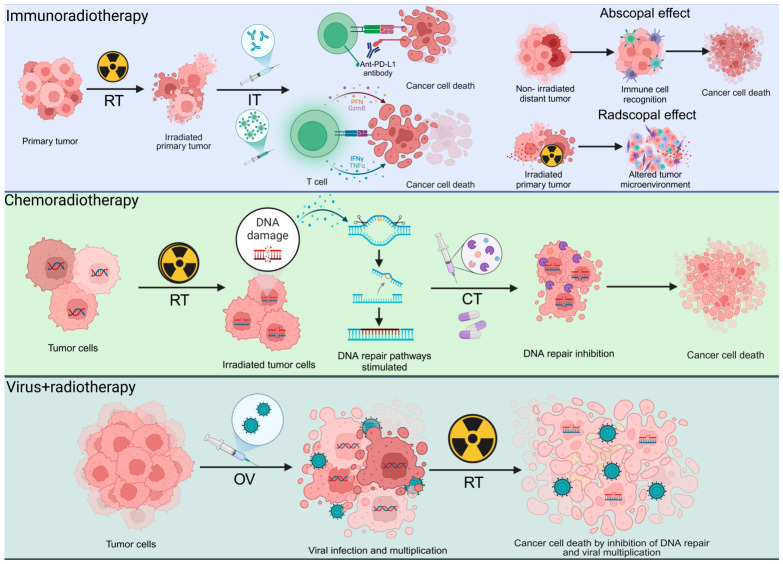
Combination approaches in radiotherapy for pediatric cancers: Immunoradiotherapy (**top panel**) combines radiotherapy with immunomodulators to enhance the antitumor immune response. Immune therapy (T-cells, checkpoint inhibitors) may increase the effectiveness of radiotherapy by preventing tumor cells from evading immune detection after being exposed to radiotherapy. Irradiated tumor cells may release tumor antigens into the bloodstream, which may trigger an immune response against distant, non-radiated tumors (abscopal effect). Irradiated tumor cells may also alter the tumor microenvironment, making it more susceptible to immune attack (radscopal effect). Chemoradiotherapy (**middle panel**) aims to increase the effectiveness of targeted agents combined with radiotherapy. Chemotherapy (PARP inhibitors) sensitizes tumor cells to the effects of radiotherapy, making them more vulnerable to DNA damage induced by radiation. Oncolytic viruses (**bottom panel**) specifically target and infect tumor cells. When combined with radiotherapy, oncolytic viruses may enhance the radiotherapy-induced cancer cell toxicity by sustaining DNA damage, increasing viral replication, and inducing an antitumor immune response.

The rationale for combination therapies is that single-agent therapies are unlikely to suffice for high-risk tumors; combinations may provide additive or synergistic effects. Several ongoing trials are investigating radiotherapy combined with targeted therapies (e.g., BRAF and MEK inhibitors), immunotherapies (e.g., PD-1 inhibitors), and novel agents like oncolytic viruses and tumor vaccines, designed to enhance tumor responses (Table 1, Figure 1).

#### 2.4.1. Strategies in Medulloblastoma

Medulloblastoma is the most common malignant brain tumor in the pediatric population [60]. The standard of care for these patients is upfront surgery followed by a combination of chemotherapy and craniospinal irradiation (CSI). CSI is followed by boost doses to the primary tumor site and to any metastatic sites [41]. Post-operatively, patients are categorized as standard-risk (66%) or high-risk (34%). Patients with standard-risk disease include those with <1.5 cm^2^ residual tumor, who are >3 years of age, and with no evidence of metastatic disease [2]. High-risk patients are those with >1.5 cm^2^ of residual tumor, evidence of metastatic disease, or anaplastic large cell histology [41]. CSI dosing is tailored based on disease risk, with standard-risk patients receiving a lower total CSI of 23.4 Gy with an involved field boost to 30.6 Gy, compared to high-risk patients who receive 36 Gy to the cranial axis with posterior fossa boost to 18–20 Gy to a total dose of 54–56 Gy. Patients who have gross spinal metastases receive additional boost radiotherapy to a total dose of 50.4 Gy [61]. Radiotherapy is avoided in children under 3 years of age due to the significant long-term effects on neurocognitive and endocrine function [2]. More recently, radiation-sparing approaches have been extended to some children younger than 6 years of age using intensive chemotherapy-based regimens, including the HeadStart protocols, which have demonstrated favorable outcomes while avoiding or delaying craniospinal irradiation in select patients. However, radiotherapy may still be necessary in cases of refractory disease, recurrence, or inadequate response to induction chemotherapy [62].

Resistance to radiotherapy remains an issue in medulloblastoma and several mechanisms may proffer resistance in these tumors. Previous work in group 3 medulloblastoma cells has shown that the loss of enhancer of zeste 2 polycomb repressive complex 2 subunit (EZH2)-mediated histone H3K27 methylation (H3K27me3) is a predictor of poor response to radiotherapy. The loss of H3K27me is associated with an epigenetic shift to acetylation (H3K27ac), which alters the transcriptional profile and causes over-activation of AKT signaling, resulting in a radiotherapy-resistant phenotype [63]. One study examined this mechanism by knocking out EZH2, leading to a loss of H3K27me3, which resulted in radiation-resistant cells. This radiation resistance was reversed with the inhibition of AKT signaling. These findings suggest there is the potential for a treatment approach guided by epigenetic profiles with regards to methylation and acetylation in these tumors [63,64].

Other studies have shown that nestin-expressing perivascular medulloblastoma stem cells are able to survive radiotherapy, promote phosphoinositide 3-kinase (PI3K)/Akt signaling, and undergo p53-dependent cell cycle arrest, subsequently re-entering the cell cycle at 72 h. This study’s findings support the role of a cancer stem cell niche in medulloblastoma radiotherapy resistance [65].

Escaping radiotherapy-induced apoptosis may be another mechanism of resistance. Buck et al. explored the potential of veliparib, a PARP inhibitor, as a radiosensitizer in MYC amplified group 3 medulloblastoma, the subgroup that is the most lethal [44,66]. They found that veliparib combined with radiotherapy exhibited effects that were additive to synergistic, enhancing DNA damage and reducing colony formation in D425GiL human medulloblastoma cells in vitro. They further showed that veliparib combined with CSI significantly improved survival in an orthotopic murine model compared to either treatment alone. In addition, there was a concomitant increase in tumor cell apoptosis seen in the specimens, confirming target engagement, and substantiating PARP inhibition as the mechanism responsible for radiosensitization. Their findings suggest that PARP inhibition holds promise for enhancing radiotherapy efficacy in medulloblastoma, warranting further investigation with more potent PARP inhibitors [44].

Overcoming tumor cell DNA repair mechanisms may enhance radiotherapy efficacy. Ferreira et al. demonstrated that a DNA repair inhibitor, AsiDNA, improved survival in medulloblastoma cell models more effectively than increasing radiation doses alone. AsiDNA’s radiosensitization was efficacious across all medulloblastoma molecular subgroups and *TP53* statuses. The effects were that of mimicking a radiotherapy dose increase. Additionally, AsiDNA administration did not increase toxicity in animals, highlighting AsiDNA as a promising candidate to enhance radiotherapy in pediatric medulloblastoma [45]. Fimepinostat, a small molecule dual histone deacetylase (HDAC)/PI3K inhibitor, has been shown to enhance radiotherapy-induced cell death from DNA damage in orthotopic models of high-grade glioma (HGG) and diffuse pontine glioma (DIPG) [13,46] and is being investigated for target validation in recurrent medulloblastoma (NCT03893487).

#### 2.4.2. Strategies in Pediatric High-Grade Glioma

Pediatric high-grade gliomas (pHGGs) are among the most fatal childhood tumors, characterized by aggressive growth and treatment resistance. pHGG includes all pediatric glioma lesions that are classified as grade 3 or grade 4 by the World Health Organization (WHO) [67]. The complex anatomic location of pHGGs, coupled with their diffuse growth patterns, frequently infiltrating adjacent healthy brain tissue, presents significant challenges for surgical removal without the risk of substantial neurologic impairment [68,69]. The efficacy of many chemotherapeutic agents to treat pHGGs is constrained by the presence of the blood–brain barrier, thereby positioning radiotherapy as the most viable option for curative treatment [70]. Children with pHGG typically undergo adjuvant radiotherapy at a dose of 50.4–60 Gy after they have recovered from surgery, usually within 8 weeks of surgical resection. Those with recurrent disease may undergo re-irradiation with 30–35 Gy, and those with a longer interval from initial radiotherapy may undergo 54–60 Gy [71].

The response of pHGG to radiotherapy appears to be related to distinct genetic mutations. Research in human specimens by Werbrouck et al. found that pHGG radioresistance is primarily linked to *TP53* mutations that disrupt DDR [72]. However, other investigators have found that a subset of wild-type *TP53* pHGG tumors may carry gain-of-function mutations in the *PPM1D* gene. *PPM1D* encodes WIP1 protein that deactivates p53, therefore *PPM1D* mutations function like *TP53* loss-of-function mutations [73]. These *PPM1D* mutant tumors show an intermediate radiosensitivity compared to *TP53* mutant and wild-type tumors. This intermediate response indicates a duality in *PPM1D*’s role: reducing radiosensitivity by inhibiting p53, but increasing it by impairing DDR mechanisms, such as inactivating ATM, ATR, and CHK1/2 proteins, and dephosphorylating H2A histone family member X (H2AX) [74].

While the exact mechanisms linking *TP53* and *PPM1D* mutations to pHGG radiosensitivity remain unclear, emerging data indicate that *PPM1D* may be a promising therapeutic target for improving radiotherapy response. For example, the PPM1D inhibitor, GSK2830371, decreased the growth of *PPM1D*-mutated diffuse intrinsic pontine glioma (DIPG) cells in vitro. It was further demonstrated that PPM1D inhibition enhanced the radiosensitivity of *PPM1D* mutant tumors in animal models [47]. These effects are believed to be due to the disruption of the homologous recombination repair pathway through p53 reactivation [74].

Deland et al. suggested that *TP53*-mutant-driven pHGG radioresistance is mediated by nuclear factor erythroid 2-related factor 2 (NRF2) pathway hyperactivation, which regulates oxidative stress responses. Since wild-type p53 represses NRF2 targets, its loss activates antioxidant pathways, reducing ROS and diminishing radiotherapy effectiveness [75]. These findings may be complicated by co-occurring mutations, such as *ATRX* loss-of-function mutations, which impair DNA repair and create an intermediate radiosensitivity phenotype [72].

Checkpoint inhibitors may be another mechanism to enhance radiotherapy response in *TP53*-mutated pHGG. Studies have shown that inhibiting checkpoint kinases like ATM and CHK1 in pHGG with *TP53* mutations enhances radiosensitivity by preventing DNA repair and forcing cells with damaged DNA into replication. Additionally, checkpoint inhibitors may reactivate quiescent glioma stem cells, making them more susceptible to radiotherapy and preventing tumor regrowth [76]. Table 2 includes a review of clinical trials utilizing combination therapy with radiation in pediatric brain tumors. In pHGG, capecitabine with radiation therapy was generally well tolerated in brainstem and pHGG in phase I trial. A phase II study is evaluating the efficacy of this regimen in children with intrinsic brainstem gliomas (PBTC-030) [77]. Lomustine, as an adjunct to temozolomide, significantly improved outcomes in children with nonmetastatic HGG who underwent field radiotherapy compared to temozolomide monotherapy [78].

#### 2.4.3. Strategies in Neuroblastoma

Neuroblastoma, the most common extracranial pediatric solid tumor, is derived from the sympathetic nervous system [1]. Radiotherapy is indicated in high-risk disease in addition to chemotherapy, surgery, and immunotherapy [79]. High-risk disease is classified based on the International Neuroblastoma Risk Group staging system (INRGSS) prior to treatment [80]. High-risk patients include those children with either localized *MYCN* amplified tumors, aged less than 18 months with *MYCN* amplified tumors, or aged more than 18 months with metastatic disease [41]. In high-risk patients, radiotherapy is generally administered to the primary tumor bed in addition to sites of metastatic disease that persist after induction chemotherapy [81]. The dose administered to these patients is typically 21.6 Gy, regardless of other factors [41]. In patients with recurrent disease that were initially classified as intermediate-risk, radiotherapy is used for disease progression after chemotherapy and second-look surgery [82].

In neuroblastoma, radiotherapy resistance has been linked to cellular prion protein (PrPC), encoded by the *PRNP* gene. Radiotherapy increases *PRNP* gene abundance resulting in an upregulation of PrPC. PrPC contributes to radioresistance by protecting cells against oxidative damage and directly activating DNA damage repair mechanisms. Targeting the PrPC pathway provides a potential strategy to counteract adaptive measures associated with radiotherapy resistance in neuroblastoma [83].

Nanotechnology-based approaches designed to directly target neuroblastoma cells have shown promise in enhancing the antitumor efficacy of radiotherapy by increasing the radiation emitted and affecting the radiotherapy-induced hypoxia in the tumor microenvironment [84]. Nanoparticles that specifically target neuroblastoma cells and incorporate elements or compounds such as gold, silver, or manganese oxide that have higher atomic numbers result in increased energy release when they are excited with external beam radiotherapy, leading to increased direct cell damage. For example, Liu and associates evaluated the effect of Fe_3_O_4_ core-TiO_2_ shell nanocomposites as potential radiosensitizers in neuroblastoma in vitro. They showed that 3,4-dihydroxyphenylacetic acid (DOPAC)-coated Fe_3_O_4_@TiO_2_ increased sensitivity to radiotherapy in SK-N-AS and SK-N-DZ human neuroblastoma cell lines compared to the bare nano-constructs or radiotherapy alone [48]. Their study may be further elaborated by utilizing nucleus-targeted nano-constructs that facilitate the targeted delivery of internal emitters more proximate to genomic DNA, potentially serving as radiosensitizers for external beam radiotherapy.

In some neuroblastoma tumors, abnormal cytoplasmic trapping of p53 disrupts their ability to respond effectively to DNA damage [48], reducing the efficacy of radiotherapy [85]. Wang and colleagues showed that nitric oxide (NO) enhances the effectiveness of low-dose ionizing radiotherapy by inducing apoptosis in neuroblastoma cells that express cytoplasmic wild-type p53. They hypothesize that the mechanism involves the NO-induced mobilization of p53 that is sequestered in the cytoplasm into the nucleus, promoting nuclear retention of p53 and leading to increased stability and transcriptional activity of p53. Their preclinical data suggest that NO donors may sensitize neuroblastoma tumor cells to p53-dependent apoptosis and improve the efficacy of radiotherapy [49].

Combining radiotherapy with a DNA repair inhibitor may prove to be another promising strategy to radiosensitize neuroblastoma cells. Dolman et al. demonstrated the importance of the catalytic subunit of DNA-dependent protein kinase (DNA-PKcs), a key protein in the non-homologous end-joining repair pathway, in neuroblastoma radiotherapy sensitivity. They identified a positive correlation between the abundance of *PRKDC*, the gene encoding DNA-PK, and poor prognosis in neuroblastoma. To explore this finding, they utilized a small molecule inhibitor of DNA-PKcs, NU7026. NU7026-induced inhibition of DNA-PKcs enhanced the effect of ionizing radiation in NGP neuroblastoma cells [50]. Another group of investigators showed that the HDAC inhibitor, vorinostat, combined with radiotherapy demonstrated additive effects on cell killing in neuroblastoma cells lines and reduced tumor volumes in animal models compared to individual treatments. This combination therapy appears to work, in part, by decreasing the levels of the DNA repair enzyme Ku-86, thereby potentiating the anticancer effects of radiotherapy [51]. Wesbuer et al. reported that higher telomerase activity was linked to reduced radiosensitivity in neuroblastoma cells. When they overexpressed telomerase activity in CHLA-90 and SK-N-SH human neuroblastoma cells, leading to elongated telomeres, the cells became resistant to radiotherapy. Conversely, suppressing telomerase activity in SK-N-SH cells using a dominant-negative mutant increased radiosensitivity [52]. Their findings align with studies showing that mice with shortened telomeres due to a mutant hTERT exhibited greater radiosensitivity [86].

#### 2.4.4. Strategies in Pediatric Rhabdomyosarcoma

Rhabdomyosarcoma (RMS) is the most common pediatric soft tissue sarcoma, accounting for 60–70% of mesenchymal tumors in children [41,87]. Patients with RMS undergo standard treatment with surgery, multi-agent chemotherapy, and radiotherapy. Patients are classified into very low, low, intermediate, and high-risk groups based on stage, group, age, and FOXO1 fusion status [88]. The need for radiotherapy depends upon this stratification with radiotherapy doses ranging from 36 to 50.4 Gy [89].

RMS tumors develop radiotherapy resistance primarily through pathways associated with DNA damage repair and oxidative stress. For example, in embryonal RMS, previous work has demonstrated that MEK/ERK pathway signaling promotes c-myc accumulation. Increased c-myc limits radiation-induced apoptosis and DNA damage while also enhancing DNA repair [90] contributing to radioresistance. Another factor contributing to radioresistance in RMS is DNA-PKcs. DNA-PK leads to radiotherapy resistance by increasing c-myc and AKT activation [91,92]. The ability of RMS cells to generate an antioxidant response by upregulating antioxidant enzymes including superoxide dismutase (SOD2), glutathione peroxidase (GPx4), and catalase (CAT) [93] provides another mechanism that contributes to radioresistance.

Factors affecting DNA damage repair, including the overexpression of the DNA methyltransferase genes, *DNMT3A* and *DNMT3B*, contribute to radioresistance in RMS. Camero and colleagues showed that silencing DNMT3A induces cellular senescence via upregulation of p16 and p21, while DNMT3B depletion causes DNA damage and impairs DNA repair mechanisms. Targeting these enzymes enhances RMS cell sensitivity to irradiation, suggesting potential for improved treatment outcomes [94]. Cai et al. explored arsenic sulfide (As_4_S_4_) as a radiosensitizer in RMS. Mechanistically, exposure to As_4_S_4_ downregulated the mRNA and protein expression of nuclear factor of activated T-cells, cytoplasmic 3, NFATC3, which induces DNA DSBs breaks via increased recombination activating gene 1 (RAG1) protein expression. In vivo experiments confirmed the combination’s efficacy in suppressing RMS growth. Clinical data from 59 patients linked NFATC3 and RAG1 expression to overall survival, with both markers serving as independent prognostic indicators [53], substantiating the clinical application of these combinations. Finally, PXD-101 (Belinostat), a hydroxamic acid-type pan-HDAC inhibitor, promoted radiotherapy-induced ROS accumulation and impaired DNA repair in RMS cells [54]. Cassandri and colleagues used MS-275 (Entinostat), a Class I and IV HDAC inhibitor, in combination with radiotherapy in PAX-FOXO1 fusion positive (FP)-RMS and fusion negative (FN)-RMS cells and showed inhibition of DNA damage repair and increased ROS formation, leading to decreased tumor cell viability and tumor growth in a radiotherapy-resistant animal model [55]. Since HDAC inhibitors may exhibit off-target effects, these investigators silenced HDAC3, a specific isoform of class-I histone deacetylase, and showed that it sensitized FP-RMS cells to radiotherapy through increased DNA damage, endoplasmic reticulum stress, apoptosis, and reduced cancer cell stemness in vitro. In vivo, HDAC3 depletion inhibited the growth of irradiated tumors compared to single approaches. These researchers further developed a novel HDAC3 inhibitor, MC4448, which demonstrated promising radiosensitizing effects, suggesting that targeting HDAC3 may be a viable strategy to overcome radiotherapy resistance in RMS [56].

Novel approaches in RMS treatment are emerging, including the use of oncolytic viral therapy as a radiosensitizing tactics. Studies have demonstrated that combining repeated doses of an engineered oncolytic Herpes Simplex virus, M002, with low-dose radiotherapy led to enhanced tumor responses in established RMS cell lines [57]. These findings were further strengthened with studies utilizing PAX-FOXO1 FP-RMS patient derived xenografts, which demonstrated that M002 combined with radiotherapy significantly enhanced tumor cell death [95].

#### 2.4.5. Strategies in Ewing Sarcoma

Ewing sarcoma (ES) is a highly aggressive tumor that arises from the bone or soft tissue in children. Survival for those with ES is 70–80% with localized tumors, and approximately 30% among those with metastatic disease. Treatment involves a combination of surgery, chemotherapy, and radiotherapy [96]. Patients with stable or improved disease following neoadjuvant chemotherapy may undergo wide excision or definitive radiotherapy. In those that undergo delayed excision, children who have positive margins receive adjuvant chemotherapy and radiotherapy. Children presenting with widely metastatic disease are given chemotherapy with consideration for palliative surgery or radiotherapy to symptomatic areas, but those with more localized metastases may be offered wide excision or definitive chemotherapy and radiotherapy. Chemotherapy with consideration of radiotherapy and surgery for local control is employed in children with progressive disease or relapse [97,98,99,100]. The recommended doses of radiotherapy for patients with ES is generally 55.8 Gy [101].

ES generally responds well to radiotherapy; however, there is a subset of patients who demonstrate a radioresistant phenotype leading to persistent or recurrent disease [102]. Research delineating the mechanisms of radioresistance in this ES subset is limited. Previous investigations have shown that insulin-like growth factor II (IGF2) is expressed in a subset of ES patients with aggressive disease and that IGF2 stimulation of ES cells led to resistance to radiotherapy. Chen et al. propose that IGF2 contributes to the radioresistant phenotype through a mechanism dependent on AKT and ERK phosphorylation [103].

Approximately 85% of ES have a chromosomal translocation t(11;22) (q24;q12) of the Ewing sarcoma breakpoint region 1 (*EWSR1*) gene on chromosome 22 and the Friend leukemia virus integration site1 (*FLI1*) gene on chromosome 11. This translocation results in a EWS:Fli1 fusion protein, which is a constitutively active transcription factor that promotes the expression of genes associated with DNA damage repair, cell cycle progression, and apoptosis inhibition [104]. Mithramycin A (MithA), an inhibitor of EWS:Fli1-mediated transcription, has previously been shown to sensitize lung and bladder cancer cell lines to radiotherapy [58]. Lin and colleagues reported that MithA radiosensitized EWS:Fli1 + ES cells in vitro and in vivo, leading to tumor cell death through apoptosis. These findings provide a role for MithA in combination with radiotherapy for ES [104]. ES also frequently harbor mutations in the tumor suppressor, TP53 [59]. Curcumin (diferuloylmethane), a dietary polyphenol derived from turmeric, has demonstrated radiosensitizing effects in other cancers, through p53-dependent and independent mechanisms. Investigators have shown that pretreatment of ES cells with curcumin augments radiotherapy-induced p21 expression and enhances DNA fragmentation, resulting in cell death through inhibition of anti-apoptotic BclXl and Mcl1 gene and protein expression [59]. These findings support further evaluation of radiosensitizing agents in ES with mutant p53 or EWS:Fli1 proteins.

#### 2.4.6. Strategies in Nephroblastoma/Wilms Tumor

Nephroblastoma, or Wilms tumor, makes up 6% of pediatric solid tumors, predominantly occurring in children under 5 years of age [41]. Radiotherapy (10.5 to 21 Gy) is indicated for stage III tumors, including abdominal stage III tumors with peritoneal dissemination or tumor rupture or spillage, as well as for residual metastatic disease following chemotherapy. Metastatic disease is treated with 10.5 to 30.6 Gy of radiotherapy depending on the metastatic site and patient age [105]. Most patients with pulmonary metastasis who have a complete response to chemotherapy will not need radiotherapy [106] unless they have high-risk histology or tumor genetics [107]. Whole-lung irradiation is recommended for recurrence if not given during initial treatment [108].

Wilms tumor is highly radiosensitive [109]. However, long-term effects of radiotherapy include gonadal dysfunction, breast cancer, and cardiovascular and pulmonary complications, underscoring the need for developing strategies that permit the use of lower doses of radiotherapy [110]. There are currently no ongoing studies addressing radiosensitization in Wilms tumor.

### 2.5. Clinical and Technical Challenges to Radiotherapy Combinations

Childhood cancer treatment poses unique clinical challenges compared to that of adult cancers, such as varied immune response, impact on growth and development, requirement to preserve future fertility, minimizing long-term effects, and focusing on quality of life beyond the standard 5-year survival rate [111].

Combining radiotherapy with chemotherapy has improved cancer survival rates since the 1980s. However, most of the current radiosensitizers are cytotoxic chemotherapies with broad side effects, limiting dose escalation. Apart from managing toxicity from combination therapies, limited translatability of the combinations to clinical settings also remains a challenge. For example, the inability of many small molecule inhibitors to cross the blood–brain barrier limits their use as radiosensitizers in childhood brain tumors. Drug delivery strategies [112] including focused ultrasound/sonoporation [113], convection enhanced delivery [114], intranasal delivery [115], intraarterial delivery [116], and nanoparticles [117], may improve the clinical applicability of these agents. Despite advances in therapeutic delivery, children treated with radiotherapy remain at risk for both acute and long-term toxicities due to the destruction of normal cells and the activation of inflammatory, thrombotic, and fibrogenic processes. Inhibiting or treating the radiotherapy-induced normal tissue damage using prophylactic radioprotectors or therapeutic agents [118] may partially overcome this issue. Studying late toxicities of rare childhood cancers is particularly challenging due to the prolonged follow-up periods necessary to accurately assess both risk and incidence. For instance, occlusive cerebrovascular disorders such as strokes and transient ischemic attacks typically emerge 20 to 25 years after CSI in medulloblastoma [119], and brain aneurysms may develop over a span of more than 30 years after treatment with CRT. The risk of cardiac mortality in some childhood cancers may rise after 25 to 30 years [120]. Additional challenges in combining immunotherapy with radiotherapy include the use of generalized radiotherapy protocols that may not account for patient-specific factors, the significant heterogeneity of tumors, and the presence of tumor-resistance mechanisms that may influence treatment responses. The synergy between radiotherapy and the immune system requires a deeper understanding at the mechanistic level. For example, the optimal radiotherapy doses, timing, and fractionation to maximize immune activation are unclear. Moreover, the sequence and dose of radiotherapy and immunotherapy remain contentious, with conflicting evidence on whether radiotherapy should precede or follow immunotherapy. There is also a lack of reliable biomarkers to predict which group of children will benefit from immunoradiotherapy, complicating personalized treatment. Therefore, more trials are needed to understand the optimal radiotherapy dosages, target selection, and rational sequencing of drug-radiation combinations in childhood cancers.

## 3. Critical View

Over recent decades, the efficacy of radiotherapy as a standalone treatment for pediatric solid tumors has markedly improved, owing to advancements in treatment planning, imaging technologies, and the development of novel irradiation (IR) techniques. Enhancing radiotherapy efficacy in pediatric cancer involves selectively targeting tumor cells while sparing normal tissues by exploiting genetic and microenvironmental differences. Strategies may include modulating DNA repair pathways to increase tumor radiosensitivity, inhibiting specific cell cycle checkpoints to limit tumor repair capacity, blocking aberrant signaling pathways to overcome radioresistance, addressing tumor hypoxia and vascular abnormalities to sensitize tumors, and protecting normal tissues using agents like radical scavengers, cytokines, or stem cells [121]. Other innovative approaches may involve the development of antibody-drug conjugates for targeted delivery of radiosensitizers directly to the tumors or repurposing existing drugs to enhance tumor sensitivity to radiotherapy by leveraging oxidative stress mechanisms. The synergy between radiotherapy and immunotherapy or chemotherapy also holds promise, but a deeper understanding of their interactions is necessary prior to universal adoption of those approaches.

The development of immunoradiotherapy that combines radiation therapy with immunomodulatory agents is a field with potential to improve pediatric solid tumor response to radiation. Recent evidence indicates that ionizing radiation may stimulate systemic antitumor immune responses [122,123] and play an active role in modulating the tumor microenvironment, particularly through immune system interactions [124]. This knowledge, combined with the expanding repertoire of immunomodulators, presents promising opportunities for innovative therapies. However, there are unique challenges, such as determining the optimal combinations, modality sequencing, and managing potential toxicities. For example, the integration of immunotherapeutic agents with radiotherapy in pediatric brain tumors faces translational barriers primarily related to the poor permeability of most immunotherapies to the blood–brain barrier. One way to overcome this issue is the use of replication competent oncolytic viruses (OVs) in combination with radiation. OVs including HSV1, adenoviruses, and polioviruses represent an alternative modality that may potentially prime antitumor immunity in situ. OVs preferentially replicate in and lyse tumor cells, such as gliomas, that exhibit permissiveness to viral infection, releasing tumor-associated antigens and danger-associated molecular patterns (DAMPs), initiating a form of immunogenic cell death that may serve as an endogenous vaccine [125]. This local antigen release enables OVs to convert the tumor into a self-sustaining source of personalized immune stimulation without requiring prior antigen identification. Moreover, OVs are generally well tolerated and amenable to repeated administration, further enhancing their utility as a tool to enhance the tumor-directed immune response in CNS malignancies. Key challenges with oncolytic virotherapy are selecting the most promising oncolytic virus platforms, balancing immune suppression and activation for optimal virus spread and anticancer response, improving preclinical models, and significantly increasing virus manufacturing yields. In addition to immunomodulating drugs and OVs, there is a need to explore adoptive cellular therapies, including CAR (chimeric antigen receptor) T-cell based approaches combined with radiotherapy in pediatric solid tumors. Historically, CAR T-cell therapies have had limited therapeutic efficacy against pediatric solid tumors [126]. To improve solid tumor response to these therapies, investigators have proposed utilizing low-dose radiation in combination. Preclinical and clinical data demonstrate that protocols where radiation therapy precedes adoptive T-cell therapy yields a better response and better infiltration with T-cells compared with protocols not involving radiation therapy in melanoma, gastric cancer, pancreatic cancer, and multiple myeloma [127]. For example, Quach and colleagues demonstrated that the use of tumor-targeted low-dose radiation in combination with CAR T-cell therapy in a murine model of mesothelioma resulted in significantly decreased tumor growth in the animals receiving combination therapy [128]. Further, in a recent study by Sodji et al., pretreatment of neuroblastoma tumors with low-dose radiation prior to CAR T-cells significantly improved infiltration of CAR T-cells into the tumors and significantly increased survival in animals bearing neuroblastoma tumors compared to those treated with radiation or CAR T-cell therapy alone [129]. There are some challenges underlying the combination of radiotherapy with CAR T-cell therapy in pediatric CNS tumors that will need to be addressed. For example, there is limited CAR T-cell trafficking across the restrictive blood–brain barrier, brain tumors exhibit a profoundly immunosuppressive tumor immune microenvironment, there may be functional exhaustion of CAR T-cells mediated by immune checkpoint upregulation, and the significant tumor heterogeneity inherent to CNS tumors may facilitate antigen escape and immune evasion [130]. There is an interest in utilizing other adoptive T-cell therapies such as natural killer (NK) cells. Unlike T-cells, ex vivo- or in vivo-activated NK cell therapy does not induce graft-versus-host disease, making them a promising allogeneic “off-the-shelf” therapeutic modality capable of eliciting antitumor responses with a reduced risk of long-term toxicity in pediatric solid tumors [131]. Additionally, low MHC-I expressing pediatric tumors are ideal targets for NK cell therapies, as reduced MHC-I impairs inhibitory signaling, rendering tumor cells vulnerable to NK cell-mediated lysis [132]. Further, it has been demonstrated that activating the killer-cell immunoglobulin-like receptor, *KIR*, gene correlates with enhanced NK cell cytotoxicity in Ewing sarcoma, rhabdomyosarcoma, neuroblastoma, lymphoma, leukemia, and brain tumors [133]. Recently, investigators have seen that radiation administered prior to CAR NK cells enhances the effects of the treatment over that of either therapy alone in hepatocellular carcinoma [134]. Similar findings have been seen in other solid tumors [135].

Recently, several precision radiation therapy methodologies have been investigated. Ultra-high-dose-rate (FLASH) radiotherapy (RT) has garnered attention for its ability to spare healthy tissues while maintaining tumor control [136]. FLASH-RT delivers high doses of radiation in extremely short time frames, and recent studies suggest it may also modulate immune responses. One proposed mechanism is that FLASH-RT reduces radiation-induced immune cell death by limiting their exposure, thereby preserving immune function and potentially enhancing responses to immune checkpoint inhibitors (ICIs) [137,138]. This synergy could be particularly valuable in pediatric solid tumors that have proven to be notoriously immunotherapy-resistant. Preclinical studies in a murine model of SHH-subtype medulloblastoma by Ni et al. demonstrated that, compared to conventional radiation therapy, FLASH-RT improved CAR T-cell infiltration and significantly prolonged survival, suggesting that FLASH-RT may potentially sensitize otherwise resistant tumors to immunotherapies [139,140]. Stereotactic body radiation therapy (SBRT) was well tolerated and demonstrated high rates of local control and improved survival outcomes in unresectable metastatic pediatric sarcomas in phase II clinical trial (NCT01763970) [141]. A phase I trial is currently investigating the safety of linear energy transfer (LET)-optimized intensity-modulated proton therapy (IMPT) in children with ependymoma (NCT03750513). Precision radiotherapy sparing healthy tissues is particularly advantageous in pediatric patients, potentially reducing adverse late effects. The noninvasive nature and convenient fractionation schedule of these approaches allow for minimal treatment breaks from systemic therapy. Finally, precision radiation therapy approaches allow for biological dose escalation that may potentially improve local control in radioresistant childhood cancers.

One of the most ambitious goals of immunoradiotherapy is to make the abscopal response a more universal and systematic one. Currently the abscopal effect is a limited, relatively rare phenomenon, with its clinical benefit restricted to a subset of patients. One explanation may be that the immunosuppressive mechanisms amplified by RT could limit a widespread systemic immune response. A phase I/II trial is currently evaluating the efficacy of a nanoparticle activated by abscopal or radscopal radiation combined with anti-PD-1/PD-L-1 in adult patients presenting with lung or liver metastases (NCT05039632). While the biological mechanisms driving the abscopal and radscopal effects may be relevant to pediatric solid tumors, preclinical studies and clinical trials are required to establish their safety, feasibility, and therapeutic efficacy in childhood cancers.

Normal tissue tolerance to radiation therapy is affected by the total and fractional radiation dose, dose rate, treatment time, radiation treatment modality, and dose distribution [142]. To balance the benefits against the risks and define quantitative evidence-based dose/volume guidelines that inform treatment planning, an international collaboration named Pediatric Normal Tissue Effects in the Clinic (PENTEC) was organized to assess normal tissue radiation dose volume response relationships for children with cancer. Their report provides methods and key findings that may be interpreted and applied in the clinic [143].

## 4. Conclusions

To improve the efficacy of radiation therapy for pediatric solid tumors, future research should focus on prioritizing precision medicine approaches to identify improved biomarkers and novel targets, leveraging the immune system as an adjunct to radiotherapy, and identifying innovative drug combinations that function in an additive or synergistic fashion with radiotherapy to optimize outcomes for pediatric cancer patients.

## Figures and Tables

**Table 1:** Preclinical studies of radiosensitizing agents in pediatric solid tumors.

Radiosensitizer	Disease Type	Model System	Key Findings/Effects
Veliparib (PARP inhibitor)	Medulloblastoma	D425GiL human cells, animal model	Enhances DNA damage, improves survival, increases apoptosis with CSI [44]
AsiDNA (DNA repair inhibitor)	Medulloblastoma	Preclinical models across all MB subtypes	Improved survival without added toxicity [45]
Fimepinostat (HDAC/PI3K inhibitor)	pHGG, DIPG	Cell lines, orthotopic mice models	Enhances cell death via DNA damage [46]
GSK2830371 (PPM1D inhibitor)	pHGG	PPM1D-mutated DIPG cells, animal models	Improves radiosensitivity by disrupting homologous recombination through p53 reactivation [47]
Fe_3_O_4_ core-TiO_2_ nanocomposites (DOPAC-coated)	Neuroblastoma	SK-N-AS and SK-N-DZ human cells	Increased cell death via direct DNA damage [48]
Nitric Oxide donors	Neuroblastoma	Cytoplasmic WT p53-expressing NB cells	Induces nuclear p53 retention, promoting apoptosis and radiosensitivity [49]
NU7026 (DNA-PKcs inhibitor)	Neuroblastoma	NGP neuroblastoma cells	Enhances radiotherapy effect by inhibiting DNA repair [50]
Vorinostat (HDAC inhibitor)	Neuroblastoma	NB cell lines, animal model	Additive cell killing with radiation and reduces tumor volume [51]
Telomerase inhibitors	Neuroblastoma	CHLA-90, SK-N-SH cells	Increased radiosensitivity [52]
Arsenic sulfide (As_4_S_4_)	Rhabdomyosarcoma	In vivo RMS models	Enhances DNA DSBs via NFATC3/RAG1, suppresses tumor growth [53]
PXD-101/Belinostat (pan-HDAC inhibitor)	Rhabdomyosarcoma	In vitro/in vivo RMS models	Promotes ROS and inhibits DNA repair [54]
MS-275/Entinostat (HDAC inhibitor)	Rhabdomyosarcoma	FP-RMS and FN-RMS models	Inhibits DNA repair, increases ROS, reduces tumor growth [55]
MC4448 (HDAC3-specific inhibitor)	Rhabdomyosarcoma	FP-RMS models	Increases DNA damage, increases apoptosis, reduces stemness [56]
M002 (Oncolytic HSV)	Rhabdomyosarcoma	RMS cell lines	Synergistic effect enhancing tumor cell death [57]
Mithramycin A	Ewing Sarcoma	EWS:Fli1+ ES cells in vitro and in vivo	Inhibits DSB repair, induces apoptosis [58]
Curcumin	Ewing Sarcoma	ES cells with mutant *TP53*	Enhances p21 expression, promotes DNA fragmentation, inhibits anti-apoptotic genes [59]

CSI—craniospinal irradiation; MB—medulloblastoma; DIPG—diffuse intrinsic pontine glioma; pHGG—pediatric high-grade glioma; WT—wild-type; NB—neuroblastoma; RMS—rhabdomyosarcoma; FP-RMS—fusion positive rhabdomyosarcoma; FN-RMS—fusion negative rhabdomyosarcoma; DSB—double-strand breaks; ES—Ewing sarcoma.

**Table 2:** Trials evaluating combination therapies in pediatric brain tumors.

NCT Number	Phase	Therapy Combination	Tumor Type(s)	Key Details	Status
NCT03605550	I	PTC596 (Unesbulin-BMI1 inhibitor) + Radiation	Diffuse Intrinsic Pontine Glioma (DIPG) and Non-Brainstem High-Grade Gliomas	Investigating the combination of a BMI1 inhibitor with upfront radiation therapy.	Active
NCT01922076	I	Adavosertib (Wee1 inhibitor) + Radiation	Newly Diagnosed DIPG	Dose-determination study of Wee1 inhibitor combined with upfront radiation therapy.	Completed
NCT02457845	I	Oncolytic Herpes virus (G207) + Radiation	Recurrent/Progressive Supratentorial Brain Tumors	Safety study of oncolytic virus therapy combined with radiation.	Completed
NCT00634231	I	AdV-tk + Radiation	Malignant Glioma Recurrent Ependymoma	Determine safety.	Completed
NCT00079339	I/II	Tipifarnib + Radiation	Newly Diagnosed DIPG	Determine MTD and efficacy.	Completed
NCT00042991	I/II	Gefitinib + Radiation	Newly Diagnosed DIPG	Evaluating safety of combining gefitinib with radiation.	Completed
NCT00006024	I	Temozolomide + Lomustine + Radiation	Newly Diagnosed Non-brainstem HGG	Evaluating safety and MTD of TMZ and Lomustine.	Completed
NCT03178032	I	Oncolytic Adenovirus (DNX-2401) + Radiation	Newly Diagnosed DIPG	Evaluating safety of oncolytic adenovirus therapy with upfront radiation.	Completed
NCT00100802	II	Temozolomide + Lomustine + Radiation	High-Grade Gliomas	Studying combination chemotherapy with radiation therapy.	Completed
NCT00357253	I	Capecitabine + Radiation	Brainstem Gliomas and High-Grade Gliomas	Dose-determination study of capecitabine combined with radiation in children with newly diagnosed glioma.	Completed
NCT00085735	III	Vincristine + Radiation + Maintenance Chemotherapy	Standard-Risk Medulloblastoma	Comparing reduced-dose craniospinal radiation with standard dose in combination with chemotherapy.	Completed
NCT00002875	III	Cyclophosphamide regimen vs. Lomustine regimen + Radiation	Newly Diagnosed Standard-Risk Medulloblastoma	Comparing cyclophosphamide regimen to lomustine regimen following radiation.	Completed
NCT00058370	NA	Radiolabeled monoclonal antibodies + Vincristine + Radiation	Standard-Risk Medulloblastoma	Determine feasibility of combining intrathecal radioimmunotherapy with radiotherapy and chemotherapy.	Completed
NCT00003203	II	Carboplatin + Radiation	Newly Diagnosed High-Risk CNS Embryonal Tumors	Determine dose and duration of carboplatin combined with radiotherapy.	Completed
NCT04482933	II	Oncolytic Herpes virus (G207) + Radiation	Recurrent/Progressive High-Grade Gliomas	Evaluating the efficacy of a single dose of oncolytic virus therapy with radiation.	Recruiting
NCT06894979	I	AZD1390 (ATM kinase inhibitor) + Radiation	Pediatric High-Grade Gliomas	Evaluating safety and recommended Phase II dose for AZD1390.	Recruiting
NCT04049669	II	Indoximod + Radiation	Relapsed or refractory Glioma, Medulloblastoma, Ependymoma, or Newly Diagnosed DIPG	Determine efficacy of combining IDO inhibitor with radiation.	Recruiting
NCT05099003	I/II	Selinexor + Radiation	Newly Diagnosed pHGG or DIPG	Evaluate toxicities, estimate MTD, and determine RP2D of Selinexor.	Suspended
NCT03416530	I	ONC201 + Radiation	H3K27M Diffuse Midline Gliomas	Dose-determination study evaluating ONC201 with upfront radiation therapy.	Terminated
NCT03690869	I/II	REGN2810 (cemiplimab) + Radiation	Newly Diagnosed pHGG, DIPG, and Recurrent pHGG	Evaluate safety, determine MTD, and assess efficacy	Terminated
NCT00278278	III	High dose Methotrexate + Radiation	Newly Diagnosed pHGG or DIPG	Determine if addition high dose MTX improves survival.	Unknown
NCT00360854	I	Elotinib + Radiation	Relapsed or Refractory Malignant Brain Tumors	Establish MTD erlotinib combined with radiation.	Unknown
NCT02681705	II	Temozolomide + Craniospinal Radiation	Medulloblastoma	Investigating the efficacy of temozolomide administered with craniospinal radiation post-surgery.	Unknown
NCT00053872	III	Vincristine + Cisplatin + Lomustine + Radiation	Medulloblastoma	Comparing different radiation regimens combined with chemotherapy post-surgery.	Unknown
NCT00276666	II	Vincristine during radiotherapy and Lomustine, Cisplatin and Vincristine after radiotherapy	Metastatic Medulloblastoma	Determine toxicity of chemotherapy combined with radiotherapy.	Unknown

DIPG—diffuse intrinsic pontine glioma; MTD—maximum tolerated dose; RP2D—recommended Phase II dose; MTX—methotrexate; pHGG—pediatric high-grade glioma; TMZ—temozolomide; CNS—central nervous system.

## Data Availability

No new data were created or analyzed in this study. Data sharing is not applicable to this article.

## Abbreviations

The following abbreviations are used in this manuscript:

ATM	Ataxia-Telangiectasia Mutated Protein
ATR	Ataxia-Telangiectasia Mutated and Rad3-Related Protein
BER	Base Excision Repair
CNS	Central Nervous System
CRT	Conformal Radiotherapy
CSI	Craniospinal Irradiation
CTLA-4	Cytotoxic T-Lymphocyte-Associated Protein-4
DDR	DNA Damage Response
DIPG	Diffuse Intrinsic Pontine Glioma
DSB	Double-Strand Break
ES/EWS	Ewing Sarcoma
FN-RMS	Fusion Negative Rhabdomyosarcoma
FP-RMS	Fusion Positive Rhabdomyosarcoma
HDAC	Histone Deacetylase
HMGB1	High-Mobility Group Box 1
HR	Homologous Recombination
ICD	Immunogenic Cell Death
IMRT	Intensity-Modulated Radiotherapy
MB	Medulloblastoma
MTD	Maximum Tolerated Dose
MTX	Methotrexate
NB	Neuroblastoma
NER	Nucleotide Excision Repair
NHEJ	Non-Homologous End Joining
PD1	Programmed Cell Death Protein 1
PD-L1	Programmed Death-Ligand 1
pHGG	Pediatric High-Grade Glioma
PI3K	Phosphoinositide 3-Kinase
RMS	Rhabdomyosarcoma
ROS	Reactive Oxygen Species
RP2D	Recommended Phase II Dose
SBRT	Stereotactic Body Radiotherapy
SHH	Sonic Hedgehog
SSB	Single-Strand Break
TMZ	Temozolomide
WT	Wild-Type
